# SNX10 Is Involved in Ovarian Cancer Cell Metastasis by Repolarizing Tumor-Associated Macrophages Through mTOR1/Lysosomes Pathway

**DOI:** 10.3390/biomedicines13051021

**Published:** 2025-04-23

**Authors:** Ranran Chai, Kewei Zheng, Ting Xu, Hui Wang, Xiaobo Cheng, Chong Lu, Yu Kang

**Affiliations:** 1 Obstetrics and Gynecology Hospital of Fudan University, Shanghai 200011, China; chairanran7664@fckyy.org.cn (R.C.); zhengkw983@163.com (K.Z.); xuting0321@126.com (T.X.); wanghui7611@fckyy.org.cn (H.W.); chengxiaobo10000@fckyy.org.cn (X.C.); 2 Shanghai Key Laboratory of Female Reproductive Endocrine Related Diseases, Shanghai 200011, China

**Keywords:** ovarian cancer, tumor associated macrophages, SNX10, lysosome, mTOR1

## Abstract

**Background:** Tumor-associated macrophages (TAMs) are prevalent in advanced ovarian cancer tissues and ascites, significantly influencing disease prognosis. However, the mechanisms driving TAM polarization and their tumor-promoting effects remain poorly understood. **Methods**: The subcellular distribution of SNX10 in ovarian cancer tissues was analyzed using single-cell datasets (GSE147082, GSE58937). The Kaplan–Meier Plotter and GEPIA2 databases were used to evaluate SNX10’s prognostic relevance. Lentivirus-mediated *SNX10* overexpression in THP-1 cells was employed in tumor cell–macrophage co-culture experiments. Transwell assays and flow cytometry assessed SNX10’s effects on ovarian cancer cell metastasis and cisplatin-induced apoptosis. RNA sequencing, Western blotting, lysosomal pH detection, lipid droplet staining, and RT-qPCR were performed to explore SNX10’s molecular mechanisms in TAM polarization and immune modulation. **Results**: SNX10 was specifically expressed in TAMs, promoting their polarization into the M2 phenotype. This enhanced the migration and invasion of ovarian cancer cell lines A2780 and A2780/CP70 while reducing cisplatin-induced apoptosis. SNX10 decreased lipid droplet content, downregulated p-mTOR1, and impaired lysosomal function in TAMs. Additionally, SNX10 differentially modulated *PD-L1* mRNA expression in platinum-sensitive and platinum-resistant ovarian cancer cells. **Conclusions**: SNX10 regulates the mTOR1/lysosome pathway in TAMs, influencing lipid metabolism and indirectly modulating ovarian cancer cell metastasis. It also alters *PD-L1* mRNA expression, suggesting a role in shaping the tumor immune microenvironment.

## 1. Introduction

Ovarian cancer is one of the poorest prognostic gynecological malignancies, with a high recurrence rate and drug resistance [1,2]. Widespread peritoneal metastasis and platinum resistance are the key treatment challenges in ovarian cancer, and the exploration of novel treatment targets is urgently needed. The tumor microenvironment (TME) of ovarian cancer is composed of tumor-associated macrophages (TAMs), dendritic cells (DCs), neutrophils, regulatory T cells (Tregs), and myeloid-derived suppressor cells (MDSCs). These immune cells interact with tumor cells through the secretion of various cytokines and inflammatory mediators, thereby promoting tumor progression and inducing immunosuppression.

TAMs, as immune cells in the tumor microenvironment, are implicated in the spread of many tumors [3]. TAMs exhibit functional plasticity, polarizing into two distinct subsets: the classically activated M1 phenotype and alternatively activated M2 phenotype. M1 macrophages predominantly secrete pro-inflammatory cytokines (IL-6, IL-12, TNF-α) that drive anti-tumor immunity. Conversely, M2-type macrophages primarily release immunosuppressive factors (IL-10, VEGF, Arg-1) that facilitate tissue remodeling, promote angiogenesis, and ultimately support tumor progression. Recent studies have demonstrated that the infiltration density of M2-type macrophages serves as a significant prognostic indicator in diverse malignancies.

In ovarian cancer tissues, macrophages are one of the main invading immune cells, predominantly in the M2 phenotype [4,5]. Macrophages make up the majority of the tumor microenvironment in ovarian cancer, implying their role in tumor growth, invasion, immune evasion, and treatment resistance [6,7]. M2-type macrophages promote epithelial–mesenchymal transition (EMT), proliferation, migration, and immune tolerance in ovarian cancer through the secretion of TGF-β, Arg-1, CCL17, CCL22, IL-10, and circTMCO3-enriched exosomes [8,9,10].

Although several studies have elucidated key molecular pathways driving macrophage polarization toward the M2 phenotype in TAMs and their functional implications in ovarian cancer [11,12], further exploration is still needed. Identifying molecules that govern the interaction of ovarian cancer cells with TAMs could lead to new therapeutic options for ovarian cancer metastasis prevention and treatment.

SNX10, a protein with a phox homology domain that binds to phosphatidylinositol on membrane structures, is found in endosomes, lysosomes, and endoplasmic reticulum. It acts as an adapter protein, facilitating signal transduction and intracellular protein trafficking [13,14,15,16]. Recent studies have established that this gene plays a critical role in regulating macrophage-mediated inflammatory responses and lipid metabolism in colitis, atherosclerosis, and intestinal cancer [17,18,19]. For instance, SNX10 deficiency induces macrophage reprogramming via the Lyn-AKT-TFEB signaling axis, attenuating foam cell formation and suppressing atherosclerosis progression. This suggests that SNX10 plays an important role in regulating macrophage function. However, it is unclear whether SNX10 regulates macrophage polarization and influences the biological activity of ovarian cancer.

In this study, we investigated the role of SNX10 in macrophage polarization and elucidated the underlying molecular mechanisms. Utilizing a co-culture system, we show that SNX10-dependent macrophage reprogramming enhances ovarian cancer metastasis, chemoresistance, and immune tolerance. These findings provide critical insights into tumor immune microenvironment regulation and offer promising therapeutic avenues for treating advanced metastatic ovarian cancers.

## 2. Materials and Methods

### 2.1. Cell Culture and Co-Culture of Tumor Cells and Macrophages

Ovarian cancer cell lines A2780 and A2780/CP70 were obtained from the Shanghai Key Laboratory of Female Reproduction Endocrine Related Diseases, Obstetrics and Gynecology Hospital, Fudan University. Human monocytic cell line THP-1 was purchased from Gasser Biotechnology Co., Ltd. (Shanghai, China). The cells were grown in DMEM media (Gibco, Carlsbad, CA, USA) containing 10% fetal bovine serum (FBS) (Excellbio, Suzhou, China) and 1% penicillin–streptomycin (NCM Biotech, Suzhou, China). The platinum-resistant A2780/CP70 cell line requires maintenance with 5 μM cisplatin to preserve its drug-resistant phenotype. The cells were maintained at 37 °C in a humidified incubator with 5% CO_2_. The 100 ng/mL phorbol 12-myristate 13-acetate (PMA) (HY-18739, MedChemExpress, Monmouth Junction, NJ, USA) was utilized for converting THP-1 cells into macrophages for 48 h, and the THP-1 cells after PMA stimulation were called M0 macrophages. The co-culture system was constructed in 6-well plates by seeding ovarian cancer cells (1 × 10^5^/well) in the transwell upper chamber, with M0 macrophages (2 × 10^5^/well) maintained in the lower chamber, in which macrophages and tumor cells were plated at a 2:1 ratio.

### 2.2. Lentivirus Transfection

Stable *SNX10* overexpressing THP-1 cell lines and the negative control were generated by using lentiviral transduction and antibiotic selection. The *SNX10*-EO plasmid (PGMLV-CMV-H_*SNX10*-3×Flag-EF1-ZsGreen1-T2A-Puro) and the negative control were purchased from Genomeditech Co., Ltd. (Shanghai, China). Lentivirus was produced using packaging plasmids (Jiangyuan Biotechnology, Nanjing, China) with the following transfection ratio: transfer plasmid/psPAX2 (packaging plasmid)/pMD2.G (envelope plasmid) = 7:5:2, and VigoFect (T001, Vigorous Biotech, Beijing, China) was used to transfect plasmids into HEK 293 T cells. After 48 h, the supernatant containing lentivirus was added to the THP-1 cells’ plates. After 72 h, 2 μg/mL puromycin (HY-B1743A, MedChemExpress, NJ, USA) was added for SNX10 overexpressing cell selection.

### 2.3. Flow Cytometry Analysis

In the transwell co-culture system, ovarian cancer cells were seeded in the upper chamber while M0 macrophages were plated in the lower chamber. When tumor cells reached 80% confluence, cisplatin was added at the IC50 concentration. After 48 h of treatment, cancer cells were harvested by trypsinization. Ovarian cancer cells were dyed with Annexin V-APC/PI apoptosis kit (Liankebio, Hangzhou, China) for 5 min in the dark at room temperature. PI was used to characterize the early stage of apoptosis, and APC was the late phase. CytoFLEX flow cytometer (Beckman 197 Coulter, Brea, CA, USA) was employed to detect the fluorescent-labeled cells, and the rate of apoptosis was measured by Flow Jo software (version 10.8.1).

### 2.4. RNA Sequencing Analysis

RNA was extracted from the *SNX10*-EO THP-1 cells, negative control THP-1 cells, and ovarian cancer cells using TRIZOL (9101, Takara, Kyoto, Japan). The mRNA sequencing and differential expression analyses were conducted at the Shanghai OE Biotech Co., Ltd. (Shanghai, China). The libraries were sequenced on a llumina Novaseq 6000 platform (Illumina, San Diego, CA, USA), and 150 bp paired-end reads were generated. About 50.30M raw reads for each sample were generated. Raw reads of fastq format were firstly processed using fastp [20], and the low-quality reads were removed to obtain the clean reads. Then, about 48.24M clean reads for each sample were retained for subsequent analyses. The clean reads were mapped to the reference genome using HISAT2 (V2.2.1) [21]. FPKM [22] of each gene was calculated, and the read counts of each gene were obtained by HTSeq-count [23].

Differential expression analysis was performed using the DESeq2 (v1.40.0) [24]. *p* value < 0.05 and log2(FC) > 2 or log2(FC) < −2 were set as the threshold for significantly differential expression genes (DEGs). Hierarchical cluster analysis of DEGs was performed using R (version 4.2.1) to demonstrate the expression pattern of genes in different groups and samples.

Based on the hypergeometric distribution, GO [25] and KEGG [26] pathway enrichment analyses of DEGs were performed to screen the significant enriched term using R (version 4.2.1), respectively. R (version 4.2.1) was used to draw the lollipop chart of the significant enrichment term.

### 2.5. Western Blotting Assay

Everolimus (1 ug/mL, HY-10218, MedChemExpress, NJ, USA), Bafilomycin A1 (500nM, HY-100558, MCE, USA), or PBS (Gibco, Carlsbad, CA, USA) was added into the supernatant of THP-1 cells and M0 macrophages for 12 h and 24 h when the cell density reached 80–90%. Cells were lysed with RIPA buffer (89901, Thermo Fisher Scientific, Waltham, MA, USA) and quantified using a BCA protein assay kit (Beyotime Biotechnology, Shanghai, China). Protein samples were boiled at 100 °C for 15 min. Then, 20 ug of proteins was separated by 10% SDS-PAGE and transferred onto PVDF membranes. Membranes were blockaded with 5% defatted milk for 1 h. The membrane was cultured with primary antibodies at 4 °C overnight and subsequently incubated with the secondary antibody (7076S, 7074S, Cell Signaling Technology, Danvers, MA, USA) for an additional 2 h. Protein bands were detected using an enhanced chemiluminescence kit (NCM Biotech, Suzhou, China), and the data were analyzed with Image J software (version 2.14.0). The primary antibodies used were SNX10 (sc-293380, Santa Cruz Biotechnology, Beijing, China, 1:1000), mTORC1 (66888-1, Proteintech, Wuhan, China, 1:1000), p-mTORC1 (5536T, Cell Signaling Technology, USA, 1:1000), and LAMP2A (66301-1-Ig, Proteintech, USA, 1:1000), and GAPDH (GB15002-100, Servicebio, Wuhan, China, 1:1000) was used as a loading control.

### 2.6. Cell Viability Assay

The half maximal inhibitory concentration (IC50) values of cisplatin in platinum-sensitive ovarian cancer cell line A2780 and platinum-resistant ovarian cancer cell line A2780/CP70 were determined by Cell Counting Kit-8 (CCK-8) assay (Beyotime Biotechnology, Shanghai, China). Ovarian cancer cells were seeded at a density of 2000 cells per well in 96-well plates. When cultures reached approximately 70% confluency, cells were treated with cisplatin (HY-17394, MedChemExpress, NJ, USA) for 48 h using the concentration gradient specified in the Supplementary Data. CCK-8 reagent (C0038, Beyotime Biotechnology, Shanghai, China) was added to each well according to the manufacturer’s protocol, followed by 3 h of incubation at 37 °C. Absorbance was then measured at 450 nm using a microplate reader.

### 2.7. Real-Time Reverse Transcription–PCR Assay

Total RNA was extracted from cells using RNA Easy Fast Tissue/Cell Kit (DP451, Tiangen Biotech, Beijing, China). RNA concentration was measured using a spectrophotometer. Next, 1 µg of RNA was reverse transcribed to cDNA using HiScript III RT SuperMix for qPCR (+gDNA wiper) (R323, Vazyme, Nanjing, China). Real-time PCR (40 cycles, annealing temperature 60 °C) was performed using Taq Pro Universal SYBR qPCR Master Mix (Q712, Vazyme, China) on a QuantStudio™ 3 System (Thermo Scientific, Waltham, MA, USA). Gene expression was normalized to GAPDH as an internal control, and the values were calculated by the 2^−ΔΔCt^ method. The primers are shown in Table 1.

### 2.8. Transwell Assays

After co-culturing with M0 macrophages for 48 h, ovarian cancer cells (1× 10^5^ cells) were collected, resuspended in serum-free medium, and seeded into the upper transwell chamber (8 μm pore size; Corning Inc., Corning, NY, USA) coated with or without Matrigel (356234, Corning, USA). Media containing 10% FBS were added to the lower chamber. Following a 24 h incubation period, the migrated cells in the lower chamber were fixed with 4% paraformaldehyde (G1101, Servicebio, Wuhan, China) and stained for 15 min with crystal violet dye (G1014, Servicebio, China). The number of migrated and invaded cells was photographed and recorded under an inverted phase contrast microscope (BX63, Olympus, Tokyo, Japan) and counted using ImageJ (version 2.14.0).

### 2.9. BODIPY Staining and Oil Red O Staining

The THP-1 cells were fixed in 4% paraformaldehyde solution and incubated in the dark with BODIPY (GP1104, Servicebio, Wuhan, China) for 30 min and DAPI (G1012, Servicebio, Wuhan, China) for 10 min and recorded under a fluorescence microscope (IX73, Olympus, Tokyo, Japan). After adding 1 μg/mL of everolimus (HY-10218, MCE, USA) or PBS to M0 macrophages for 12 h and 24 h, the cell culture solution was sucked up from the 6-well plate, 4% paraformaldehyde fixative (30525-89-4, Hesse, Germany) was added for 10 min, and then it was washed twice with PBS. Then, 1.5 ml of 60% isopropanol (67-63-0, Aladdin, Beijing, China) was added for 15–20 s, it was allowed to dry slightly, and then oil red O working solution (mixed with distilled water with 6:4) was added for 30 min in the dark. Then, 60% isopropanol was added again for 3–5 s, it was washed with distilled water 3 times, hematoxylin staining solution (C0107, Beyotime Biotechnology, Shanghai, China) was added for cell nucleus staining, and then the solution was observed under an inverted phase contrast microscope (BX63, Olympus, Tokyo, Japan).

### 2.10. Lysosome pH Detection

The function and quantity of lysosomes in the THP-1 cells were detected by Lysosomal Acidic pH Detection Kit-Green/Deep Red (L268, DOJINDO, Kumamoto Prefecture, Japan). The kit includes two reagents: PHLys Green and Lysprime Deep Red. PHLys Green is pH-dependent and can detect the function of lysosomes, while Lysprime Deep Red is pH-independent and used to detect the number of lysosomes. The SNX10-EO and negative control THP-1 cells were washed with HBSS twice, and then PHLys Green/Lysprime Deep Red working solution was added. After 30 min of incubation, the dyed cells were washed by HBSS twice and observed with a fluorescence microscope (IX73, Olympus, Tokyo, Japan).

### 2.11. Database Analysis

For online survival analysis and protein analysis, the Kaplan–Meier Plotter database (https://kmplot.com/analysis/index.php?p=service&cancer=ovar (accessed on 4 February 2024)) and the GEPIA2 database (http://gepia2.cancer-pku.cn/#index (accessed on 4 February 2024)) were used. For analyzing the relationship between immune cells and ovarian cancer online, TIMER 2.0 (http://timer.cistrome.org) and TISIDB (http://cis.hku.hk/TISIDB/index.php (accessed on 4 February 2024)) were used. We used single-cell datasets GSE147082 and GSE158937 to analyze the cell distribution of SNX10 in ovarian cancer tissues [27,28].

### 2.12. Statistical Analysis

GraphPad Prism software (version 7.0, San Diego, CA, USA) was utilized to analyze the data. Data were expressed as means ±SD from three individual repeats. Student’s *t*-test was applied for comparison between the two groups. The comparison among multiple groups was analyzed by one-way ANOVA. *p* < 0.05 represented the statistical difference.

## 3. Results

### 3.1. SNX10 Is Highly Expressed in Macrophages of Ovarian Cancer Tissue and Affects Prognosis

**Figure 1:**
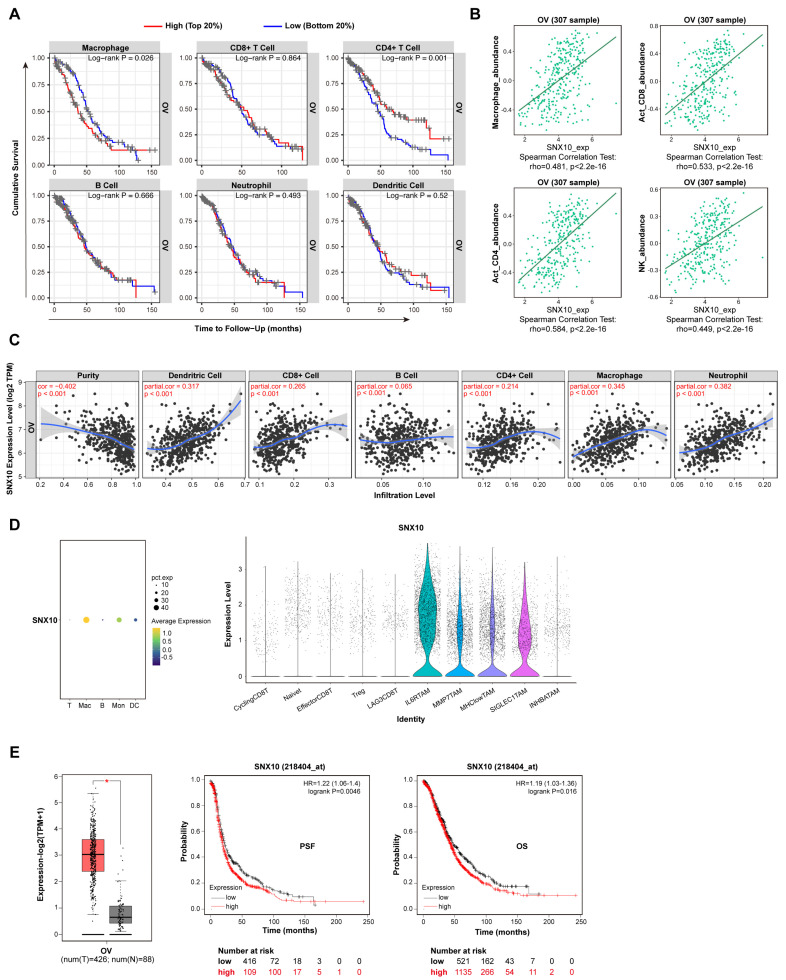
SNX10 is predominantly expressed in macrophages in ovarian cancer tumor tissues and exhibits a negative correlation with ovarian cancer prognosis. (**A**) Using TIMER 2.0 online databases, analyze the correlation between immune cell proportion in ovarian cancer tissues and prognosis. (**B**,**C**) TISIDB online and the TIMER 2.0 database analyze the correlation between SNX10 expression level and immune cells in ovarian cancer tumor tissues. (**D**) Analysis of the distribution of SNX10 in subcellular populations of ovarian cancer tissues using the GSE147082 and GSE158937 single-cell datasets. (**E**) Analysis of the link between SNX10 expression in ovarian cancer and progression-free survival (PFS) or overall survival (OS) through the GEPIA2 database (the red columns = ovarian cancer; the grey columns = normal ovary tissue) and Kaplan database. * *p* < 0.05.

TAMs play a well-established role in modulating tumor cell behavior within the tumor microenvironment. After searching the online database TIMER2.0 (Tumor Immune Estimation Resource) and TISIDB (an integrated repository portal for tumor-immune system interactions), it was discovered that the proportion of TAM infiltration is negatively correlated with prognosis, whereas CD4+T cells are positively correlated with prognosis in ovarian cancer (Figure 1A). SNX10, previously implicated in macrophage-related pathologies such as fatty liver disease and atherosclerosis [19,29], was found to be significantly overexpressed in ovarian cancer. We discovered that SNX10 expression was positively linked with the infiltration of macrophages, neutrophils, dendritic cells, CD4+T cells, and CD8+T cells in ovarian tumor tissue (Figure 1B,C). Next, by evaluating the GSE147082 and GSE158937 ovarian cancer single-cell datasets, it was shown that SNX10 is primarily expressed in tumor-associated macrophages (TAMs) rather than tumor cells (Figure 1D). Searching the GEPIA2 and Kaplan–Meier Plotter databases revealed that SNX10 is substantially expressed in ovarian cancer and has a negative correlation with progression-free survival (PFS) or overall survival (OS) of ovarian cancer (Figure 1E). These results indicate that SNX10 is mostly expressed in tumor-associated macrophages in ovarian cancer tissue, and we hypothesize that SNX10 influences macrophages to regulate tumor cell biology.

### 3.2. Overexpression of SNX10 in Macrophages Increases the Migration and Invasion Ability of Ovarian Cancer Cells

**Figure 2:**
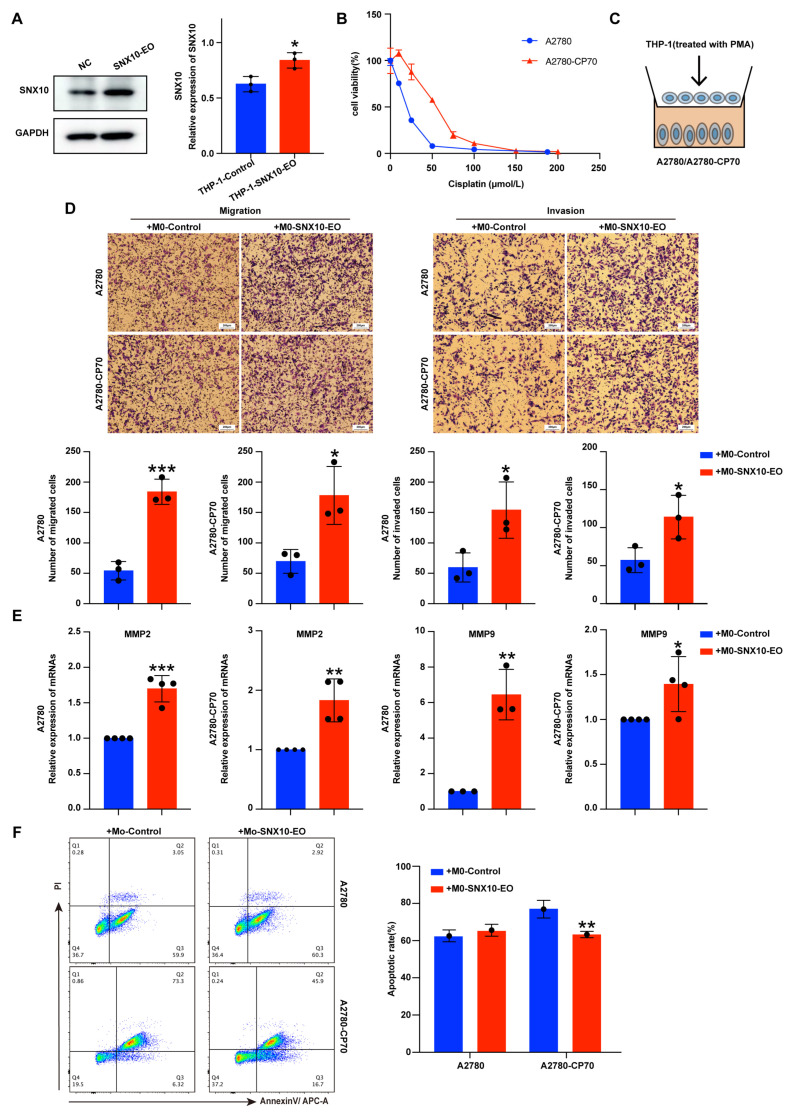
Overexpression of SNX10 in THP-1 cells promotes ovarian cancer cell invasion and migration. (**A**) Establishment of stable overexpression of *SNX10* in the THP-1 cell line; the column diagrams on the right display the statistical differences in protein expression levels in the two groups. (**B**) The CCK8 method was used to detect the cisplatin sensitivity of ovarian cancer cell lines A2780 (platinum-sensitive) and A2780/CP70 (platinum-resistant) (IC50: 3.15 μM vs. 46.7 μM, respectively). (**C**) The diagrammatic sketch of cell co-culture system. (**D**) Transwell assays were used to assess the migratory and invasive properties of A2780 and A2780/CP70 cells co-cultured with M0-control and M0-*SNX10*-EO. (**E**) RT-qPCR was utilized to evaluate the gene expression levels of migration-related markers (*MMP2, MMP9*) in ovarian cancer cells. (**F**) The apoptotic rate of cisplatin on ovarian cancer cells after co-culturing with M0-control and M0-*SNX10*-EO groups for 48 h was detected by flow cytometry; the column diagram on the right displays the statistical analysis of the apoptosis rate of ovarian cancer cells. * *p* < 0.05; ** *p* < 0.01; *** *p* < 0.001. All experiments were repeated at least three times.

Based on the finding that SNX10 is mostly expressed in TAMs in ovarian cancer, we aim to investigate its functional role in ovarian cancer biology via macrophages. First, we overexpressed *SNX10* in THP-1 cells via lentiviral infection and verified it by Western blotting (Figure 2A). Platinum-sensitive ovarian cancer cells A2780 and platinum-resistant ovarian cancer cells A2780/CP70 were selected. The CCK8 method was used to obtain their cisplatin IC50 value. For cisplatin, the IC50 of A2780 is 3.15 μM, while the IC50 of A2780/CP70 is 46.7 μM (Figure 2B). We examined the migration and invasion abilities of ovarian cancer cells after 48 h of co-culturing with M0 macrophages (Figure 2C). The results revealed that co-culturing ovarian cancer cells with M0 macrophages, which *SNX10* overexpressed (M0-*SXN10*-EO macrophages), improves their metastatic and invasive abilities (Figure 2D). This pro-metastatic phenotype was further supported by elevated *MMP2/MMP9* expression (Figure 2E). Notably, *SNX10*-overexpressing macrophages also promoted platinum resistance. Flow cytometry analysis revealed that co-culturing with M0-*SNX10*-EO macrophages markedly reduced apoptosis in A2780/CP70 cells (vs. untreated controls) when exposed to their respective IC50 doses of cisplatin for 48 h, while no significant effect was observed in A2780 cells (Figure 2F). The above data demonstrate that SNX10 overexpression in macrophages drives ovarian cancer cell aggressiveness by enhancing metastatic potential and platinum resistance.

### 3.3. SNX10 Overexpression Promotes M2-like Macrophage Polarization

**Figure 3:**
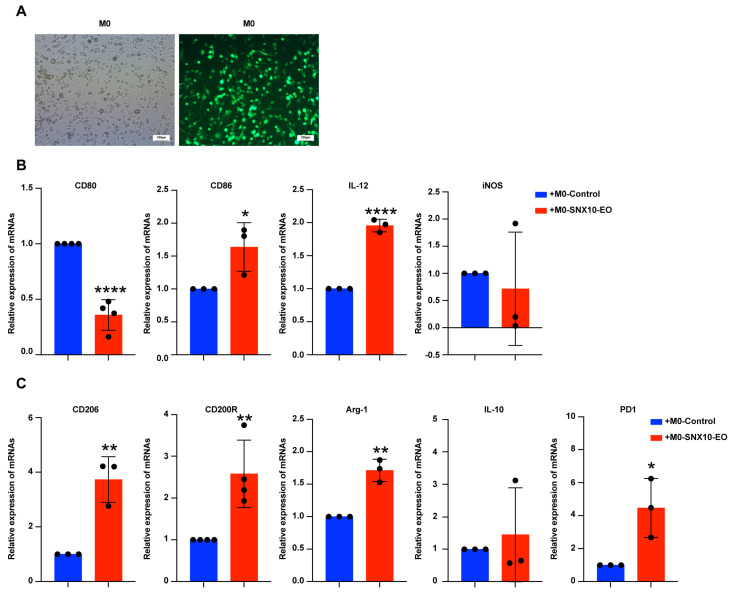
SNX10 regulates the expression of macrophage markers. (**A**) Fluorescence microscopy was used to detect the cell morphology of THP-1 cells differentiated into M0 macrophages after being stimulated by PMA. RT-qPCR was utilized to measure the relative mRNA expression levels of M1 macrophage markers (*CD80*, *CD86*, *iL-12*, *iNOS*) (**B**) and M2 macrophage markers (*CD206*, *CD200R*, *Arg-1*, *il-10*, *PD1*) (**C**) in the M0-control and M0-SNX10-EO groups. * *p* < 0.05; ** *p* < 0.01; **** *p* < 0.0001. All experiments were repeated at least three times.

Because M0-*SNX10*-EO macrophages increase ovarian cancer invasive behavior, we investigated whether SNX10 overexpression impacts macrophage differentiation into M1 or M2 types. Using RT-qPCR technology, we detected the change in M1 marker (*CD80*, *CD86*, *il-12*, *iNOS*) and M2 marker (*CD206*, *CD200R*, *Arg-1*, *il-10*) expression in M0-*SNX10*-EO macrophages and M0-control macrophage. Firstly, THP-1 cells were treated with PMA for 48 h to induce differentiation into M0 macrophages. Fluorescence microscopy reveals that M0 macrophages have unique antennas (Figure 3A). Compared to the M0-control macrophages, *CD80* mRNA expression, an M1 macrophage marker, were dramatically reduced in M0-*SNX10*-EO macrophages, but *CD86* and *il-12* increased significantly. *iNOS* decreased but not significantly. M2-associated marker mRNA expression (*CD206*, *CD200R*, *Arg-1*) was significantly increased except for *IL-10* (Figure 3B,C). Moreover, *PD1* mRNA expression increased in M0-*SNX10*-EO macrophages (Figure 3B,C). These results indicate that M0-*SNX10*-EO macrophages showed an M2 phenotype, which has a pro-cancer effect.

### 3.4. SNX10 Regulates Macrophage Polarization Through mTORC1-Mediated Lipid Metabolic Reprogramming

**Figure 4:**
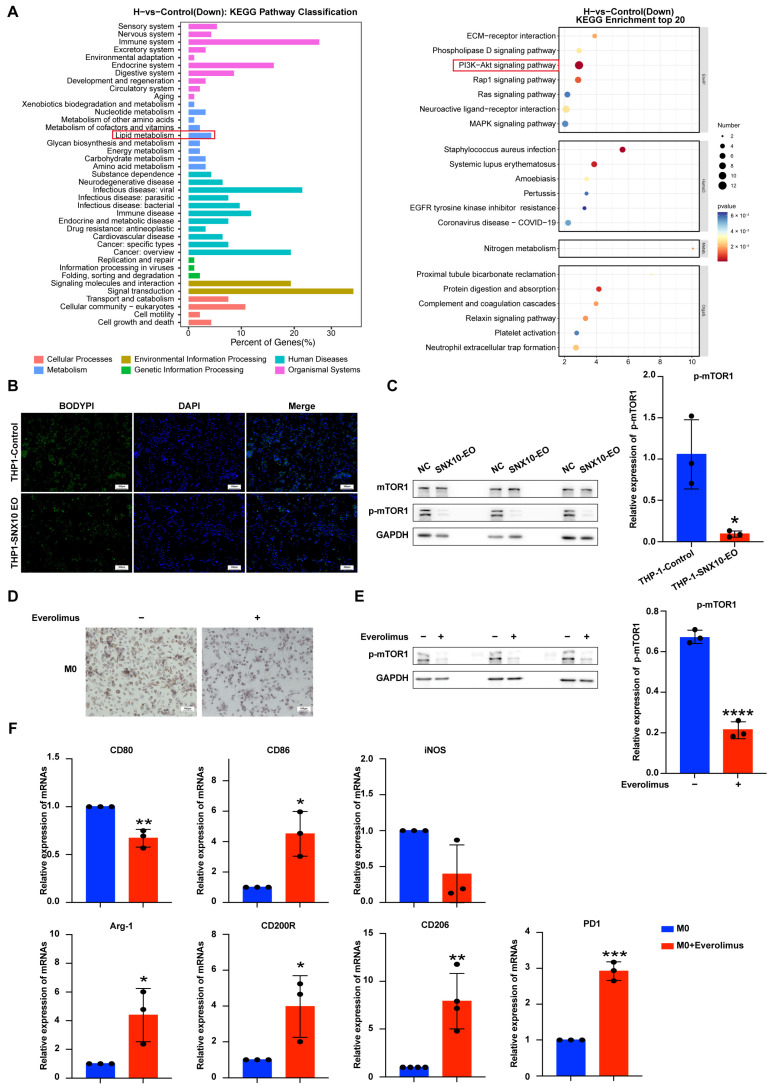
SNX10 regulates macrophage polarization through mTORC1-dependent lipid metabolic reprogramming. (**A**) KEGG signaling pathway enrichment analysis on downregulated genes (top 20 were listed) in THP-1 cells. (**B**) Lipid droplet contents were examined using BODIPY staining in THP-1 cells. (**C**) The expression of p-mTORC1 proteins in THP-1 cells was assessed by Western blotting under conditions with and without SNX10 overexpression. The column diagram on the right displays the expression levels of proteins in each group statistically analyzed by gray scanning. (**D**) Oil red staining was performed to examine lipid droplets in M0 macrophages after adding everolimus or PBS for 24 h. (**E**) The expression of p-mTORC1 proteins in M0 macrophages was analyzed by Western blotting after adding everolimus or PBS for 24 h; the column diagram on the right displays the expression levels of proteins in each group statistically analyzed by gray scanning. (**F**) RT-qPCR was utilized to measure the relative mRNA expression levels of M1 macrophage markers (*CD80*, *CD86*, *iNOS*) and M2 macrophage markers (*CD200R*, *CD206*, *Arg-1*, *PD1*) in the M0 macrophages after adding everolimus or PBS for 24 h. * *p* < 0.05; ** *p* < 0.01; *** *p* < 0.001, **** *p* < 0.0001. All experiments were repeated at least three times.

To elucidate the mechanism underlying SNX10-mediated macrophage polarization, we performed RNA-seq analysis followed by KEGG pathway enrichment. The results showed that SNX10 overexpression significantly downregulated genes involved in immune response and signal transduction, particularly those associated with lipid metabolism and the PI3K/Akt pathway (Figure 4A). Consistent with the transcriptomic findings, BODIPY staining revealed a marked reduction in lipid droplet accumulation in both THP-1 cells and M0 macrophages upon SNX10 overexpression (Figure 4B). Mechanistically, SNX10 overexpression selectively suppressed mTORC1 phosphorylation (p-mTORC1) without altering total mTORC1 protein levels (Figure 4C). To confirm the link between mTORC1 and lipid droplets, adding everolimus (an mTORC1 inhibitor) to M0 macrophage culture medium for 24 h resulted in a significant reduction in lipid droplets (Figure 4D,E). Furthermore, we found that M0 macrophages differentiated into M2 type after being treated with everolimus (Figure 4F). The data above demonstrate that SNX10 drives macrophage polarization toward an M2-like phenotype by modulating mTORC1-dependent lipid droplet reduction.

### 3.5. Overexpression of SNX10 Decreases the Number of Lysosomes and Induces Acidification Disorders to Promote M2 Macrophage Polarization

**Figure 5:**
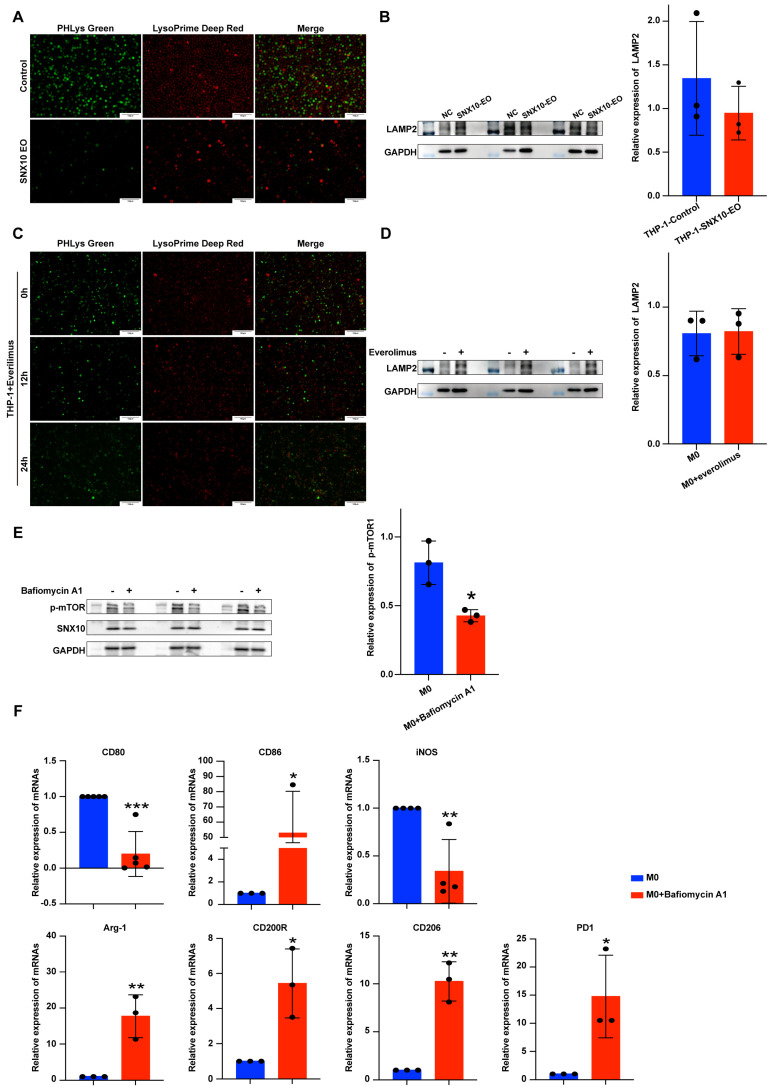
Overexpression of SNX10 decreases the number of lysosomes and induces acidification disorders to promote M2 macrophage polarization. (**A**) The lysosomal pH and amount in THP-1cells with SNX10 overexpressed or control were detected using Lysosomal Acidic pH Detection Kit-Green/Deep Red with a fluorescence microscope. Green fluorescence indicates the pH of lysosomes, while red fluorescence indicates lysosomal amount. (**B**) The expression of LAMP2A proteins in the two THP-1 cell groups was analyzed by Western blotting. The column diagram on the right displays the expression levels of proteins in each group statistically analyzed by gray scanning. (**C**) Fluorescence microscopy was used to detect pH and lysosome amount in THP-1 cells 12 and 24 h following the addition of everolimus or PBS, respectively. (**D**) The expression of LAMP2A proteins in the M0 macrophages after the addition of everolimus or PBS was analyzed by Western blotting; the column diagram on the right displays the expression levels of proteins in each group statistically analyzed by gray scanning. (**E**) After adding BafiomycinA1 or PBS for 12 h, the expression of p-mTORC1 proteins in THP-1 cells was analyzed by Western blotting. The column diagram on the right displays the expression levels of proteins in each group statistically analyzed by gray scanning. (**F**) RT-qPCR was utilized to measure the relative mRNA expression levels of M1 macrophage markers (CD80, CD86, iNOS) and M2 macrophage markers (CD200R, CD206, Arg-1, PD1) in the M0 macrophages after adding BafiomycinA1 or PBS for 12 h. * *p* < 0.05; ** *p* < 0.01; *** *p* < 0.001. All experiments were repeated at least three times.

Previous research has shown that SNX10 regulates lysosomal function in both intestinal inflammation and intestinal tumors [18]. Lysosomes are involved in the degradation of lipid droplets [30,31]. The Lysosomal Acidic pH Detection Kit-Green/Deep Red was used to assess the function and number of lysosomes in THP-1 cells. The red fluorescence represents lysosome number, while the green fluorescence shows lysosomal pH. The results showed that when SNX10 was overexpressed, both red and green fluorescence dropped, implying that the quantity and function of macrophage lysosomes were dramatically reduced (Figure 5A). LAMP2A, one of the primary lysosomal membrane proteins, did not show any significant alterations when SNX10 was overexpressed (Figure 5B). To investigate the link between SNX10, mTORC1, and lysosomes, firstly, we found that the pH and quantity of lysosomes significantly decreased when adding everolimus for 12 h and 24 h, but the expression of LAMP2A did not significantly change (Figure 5C,D). Next, Bafilomycin A1 (a lysosomal proton pump inhibitor) was added to the supernatant of THP-1 cells and incubated for 12 h. Western blotting results showed that p-mTORC1 protein expression decreased significantly except for SNX10 (Figure 5E). Finally, we used RT-qPCR to evaluate changes in M1 and M2 markers of M0-type macrophages following the addition of Bafilomycin A1, and the results revealed that the macrophages differentiated into M2 macrophages (Figure 5F). The results above show that mTORC1 phosphorylation level and lysosomal function interact with one another, and SNX10 is positioned upstream of the two.

### 3.6. SNX10-Overexpressing Macrophages Modulate PD-L1 Expression and Lipid Metabolism in Ovarian Cancer Cells

**Figure 6:**
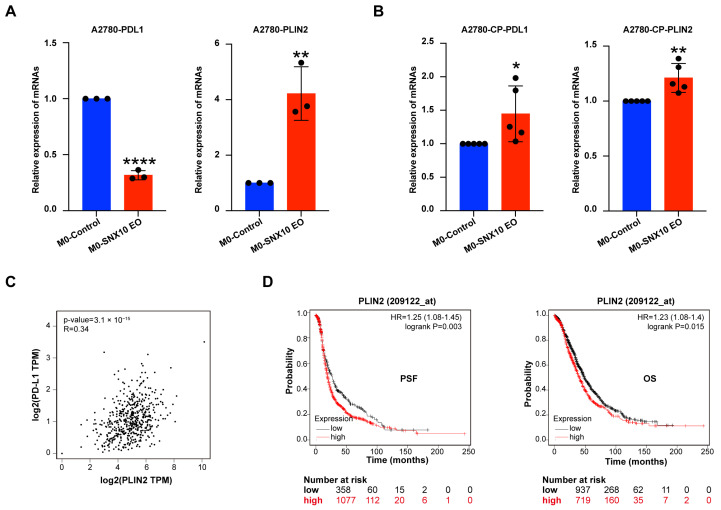
The effects on the *PD-L1* expression in ovarian cancer cells after co-culturing with M0-SNX10-EO macrophages. (**A**,**B**) RT-qPCR was utilized to measure the relative mRNA expression levels of *PD-L1* in A2780 and A2780/CP70 cells after being co-cultured with M0-control and M0-*SNX10* EO groups. (**C**) Correlation analysis of mRNA expression levels of *PD-L1* and *PLIN2* in ovarian cancer cells by the GEPIA2 database. (**D**) The Kaplan–Meier Plotter database analyzes the relationship between the expression of PLIN2 and the prognosis of ovarian cancer patients. * *p* < 0.05, ** *p* < 0.01; **** *p* < 0.0001.

Given the established role of M2 macrophages in promoting immune tolerance, we examined the *PD-L1* mRNA level in these cells. The results showed that the mRNA expression level of *PD-L1* increased in the platinum-resistant cell line A2780/CP70 but decreased in the platinum-sensitive cell line 2780. Since SNX10 causes macrophage lipid metabolism dysfunction, in order to explore whether it also affects the lipid content in ovarian cancer cells, the lipid droplet coating protein (*PLIN2*) mRNA expression in tumor cells was investigated. We discovered that *PLIN2* mRNA levels were higher in both A2780 and A2780/cp70 (Figure 6A,B). Analyzing the GEPIA2 database, we discovered that PLIN2 expression is positively linked with PD-L1 (R = 0.34) (Figure 6C). Searching the Kaplan–Meier Plotter database, it was found that PLIN2 was negatively correlated with PFS and OS in ovarian cancer (Figure 6D).

## 4. Discussion

Immune checkpoint inhibitors exhibit significant anti-tumor effects in various types of tumors. Ovarian cancer, however, is known as a “cold tumor” due to its poor response to immune checkpoint inhibitors. The development of single-cell technology has enabled a better understanding of the cellular composition of tumor tissues. It has been discovered that T cells, macrophages, NK cells, tumor-associated fibroblasts, and tumor cells within the tumor microenvironment interact with each other, participating in the regulation of immune tolerance in tumor cells and influencing tumor growth, invasion, and metastasis [32]. Tumor-associated macrophages (TAMs) are categorized into two types: M1 TAMs, which have anti-cancer and pro-inflammatory properties, and M2 TAMs, which have the opposite impact. By producing cytokines, such as interferon, TNF, IL-10, IL-12, and VEGF, controlling the PD-1/PD-L1 signaling axis, and suppressing anti-tumor immune responses, M2 TAMs attract immunosuppressive cells and cause the depletion of cytotoxic T cells [33,34]. Research has shown that M2 macrophages are one of the primary infiltrators of ovarian cancer tissues, and the prognosis of ovarian cancer is inversely connected with the amount of infiltrated M2 macrophages [35]. Tumor-associated macrophages (TAMs) have been identified as one of the key immune cells in the tumor microenvironment in ovarian cancer tumor tissues and malignant ascites. However, further research is still needed to explore how M2 macrophages contribute to ovarian cancer cells.

SNX10 is a membrane-trafficking protein containing a phosphoinositide-binding PX domain, localized to endosomes, lysosomes, and the endoplasmic reticulum [15]. It has roles in the lysosome/endosome pathway, signal transduction, and intracellular protein transport and participates in the occurrence and development of diseases such as osteolysis, gastroenteritis, atherosclerosis, and colorectal cancer [16,19,36], but its function in ovarian cancer was previously unknown. Our findings indicate that SNX10 is highly expressed in epithelial ovarian cancer and negatively correlated with prognosis. Single-cell analysis of ovarian cancer tumor tissues indicated that SNX10 is mainly expressed in TAMs. This suggests that SNX10 influences the prognosis of ovarian cancer via TAMs.

SNX10 overexpression dramatically increased the expression of the M2 marker (*Arg-1*, *CD206*, *CD200R*), while for the M1 marker, *CD80* mRNA expression significantly decreased, and the expression of *IL-12* and *CD86* significantly increased. Although *CD86* was previously thought to be an M1 marker, it has now been discovered that in the M2b subtype, *CD86* and *CD206* expression increased simultaneously [37]. Therefore, these findings demonstrate that SNX10 drives M0 macrophages toward an M2-dominant polarization state.

Lipid reprogramming of TMAs regulates their polarization and is closely associated with tumor immune suppression [38,39,40]. The three main components of lipid metabolism include fatty acids, cholesterol, and lipid droplets, and they have biological roles as signaling molecules, energy storage facilities, and components of cell membranes [41]. It has been reported that M1 TAMs prefer glycolysis for energy supply, while M2 TAMs rely on fatty acid oxidation for energy. TAMs in various cancers accumulate lipids and have increased fatty acid metabolism, which promotes malignancy and causes treatment resistance [42,43,44]. However, it has been found that the intracellular lipids of TAMs are reduced in some cancers. Cholesterol-25-hydroxylase is highly expressed in immunosuppressive macrophage subpopulations, promoting cholesterol breakdown and reducing cholesterol accumulation, which is negatively correlated with the survival rate of patients with various tumors [45]. The outward migration of intracellular cholesterol promotes the conversion of TAMs into M2 type [46,47,48].

Recent studies have shown that SNX10 is a critical regulatory protein for lipid metabolism in several diseases [19,29]. SNX10 deficiency enhances the development of alcoholic fatty liver by decreasing the degradation of LAMP2A/PLIN2 in the liver [29]. In cardiovascular diseases [16,18], increased SNX10 expression stimulates lipid accumulation in macrophages via the AKT/TFEB signaling pathway, which contributes to atherosclerosis. In our study, SNX10 overexpression or the addition of the mTORC1 inhibitor everolimus significantly reduced p-mTORC1 protein expression and lipid droplet content in M0 macrophages, and both of them polarized macrophages to M2 type, indicating that abnormal SNX10 expression could lead to lipid droplet metabolism disorder in macrophages, abnormal transmission of the mTORC1 signaling pathway, and regulation of macrophage polarization.

SNX10 regulates generation of endoplasmic reticulum and lysosomal function in the occurrence and development of various diseases [49,50,51]. Lysosomal pH, which is predominantly regulated by vacuolar H+-transporting ATPase (V-ATPase), is critical for lysosomal function stability [52,53,54]. mTORC1 is recruited to lysosomal membranes and activated through multiple pathways, which can regulate intracellular metabolism and autophagy [55,56,57]. It has been reported that the V-ATPase complex on lysosomes can regulate mTORC1 activity by modulating lysosomal pH [58]. The membrane contact between the endoplasmic reticulum and lysosomes serves as a hub for activating cholesterol-dependent mTORC1 signaling [59]. mTORC1 plays a role in lipid metabolism after activation on lysosomes, mainly promoting lipid synthesis and inhibiting lipolysis [55]. We speculate that there is a link between SNX10, mTORC1, and lysosomes in macrophages. The results demonstrated that regardless of whether SNX10 is overexpressed or the addition of everolimus (the mTORC1 inhibitor) to THP-1 cells, the number and pH value of lysosomes is reduced in THP-1 cells.

Lysosome-associated membrane protein 2A (LAMP2A), a crucial protein in chaperone-mediated autophagy (CMA), serves as a key indicator of lysosomal structural integrity and functional activity [60,61]. LAMP2A interacts with the lysosomal cation channel TMEM175 to modulate proton conduction, thereby regulating intra-lysosomal pH homeostasis [62]. The data indicated that LAMP2A protein expression did not change significantly when lysosomal pH was disrupted by SNX10 overexpression or by everolimus. When Bafiomycin A1 (a lysosomal proton pump inhibitor) was added, M0 macrophages polarized to M2, and p-mTORC1 expression was drastically reduced. According to the above results, we speculate that both mTORC1 phosphorylation level and lysosomal pH are regulated by SNX10, which ultimately affects the content of lipid droplets in macrophages and the polarization direction of macrophages but does not affect LAMP2A protein expression.

For tumor cells, we examined their immune tolerance status after co-culturing. The data revealed that the expression levels of *PD-L1* mRNA in platinum-resistant ovarian cancer cell A2780/CP70 increased significantly, but in platinum-sensitive ovarian cancer cells they decreased. High PD-L1 expression is involved in the proliferation and spread of ovarian cancer cells, altering the prognosis of ovarian cancer, according to previous studies [63]. Platinum-resistant ovarian cancer has greater PD-L1 expression levels, which are adversely linked with prognosis [64]. Studies have revealed that in glioblastoma, TAMs promote tumor progression by transferring lipids to cancer cells or secreting cytokines, thereby modulating the lipid metabolism of tumor cells [65].

Studies have found that the short-chain fatty acid acetic acid can promote the metabolic reprogramming of tumor cells and upregulate the expression of PD-L1 [66]. Itaconic acid, a metabolite of macrophages, can prolong the half-life of PD-L1 and enhance its stability after being transported to tumor cells [67]. To explore whether tumor cells have lipid metabolism disorders after being co-cultured with M0-*SNX10*-EO macrophages, we evaluated *PLIN2* mRNA levels in the ovarian cancer cells A2780 and A2780/CP70 and observed significant upregulation in both cell lines. PLIN2 expression had a negative correlation with ovarian cancer prognosis as assessed by the Kaplan–Meier Plotter database. The correlation between *PLIN2* and *PD-L1* was examined using the online databases GEPIA2, and it was discovered that there was a positive correlation (R = 0.34).

In this study, there are still some shortcomings. For example, the specific molecular mechanism underlying the reduction in lipid droplet content of macrophages has yet to be explored. It is unknown what factors are involved in the signal transmission between TAMs and tumor cells. Further studies are required to elucidate how SNX10-overexpressing macrophages modulate tumor cell behavior.

Based on the above data, overexpression of SNX10 promotes macrophage polarization to M2 type by the SNX10/mTORC1/lysosome pathway and regulates PD-L1 expression in ovarian cancer cells. SNX10 could be developed as a promising new biological target for ovarian cancer therapy.

## 5. Conclusions

In summary, we have demonstrated that SNX10 is specifically expressed in tumor-associated macrophages (TAMs) within ovarian cancer tissues. Its overexpression promotes the polarization of TAMs into the M2 phenotype, enhances the metastatic potential of ovarian cancer cells, and modulates *PD-L1* mRNA expression in tumor cells. Furthermore, our research revealed that SNX10 regulates TAM polarization by influencing p-mTOR1 expression levels and lysosomal function. These findings suggest that the regulatory mechanisms of TAMs in ovarian cancer represent a promising new therapeutic target, requiring further in-depth research for exploration.

## Figures and Tables

**Table 1:** Primers for real-time polymerase chain reaction (PCR).

Gene Name	Primer Sequences (5′ to 3′)
*PD-L1*	Forward: GCATTTGCTGAACGCATTTACT Reverse: ATTTCCCAGGGAGAGCTGGT
*PD1*	Forward: CCAAGGCGCAGATCAAAGAG Reverse: TGGCTCCTATTGTCCCTCGTG
*CD80*	Forward: GGGGAAATGCGCCTCTCTG Reverse: GTGGATTTAGTTTCACAGCTTGC
*iNOS*	Forward: TCCCGAGTCAGAGTCACCAT Reverse: CATGCAGACAACCTTGGGGT
*CD206*	Forward: GTGATGGGACCCCTGTAACG Reverse: CTGCCCAGTACCCATCCTTG
*IL-12A*	Forward: AAAGATAAAACCAGCACAGTGGAG Reverse: CCAGGCAACTCCCATTAGTTAT
*IL-10*	Forward: AGGGCACCCAGTCTGAGAAC Reverse: GGCAACCCAGGTAACCCTTAAAGT
*VEGF*	Forward: TAAGTCCTGGAGCGTTCCCT Reverse: ACGCGAGTCTGTGTTTTTGC
*PLIN2*	Forward: ACAACCGAGTGTGGTGACTC Reverse: TCTTCACACCGTTCTCTGCC
*MMP2*	Forward: AAGTATGGGAACGCCGATGG Reverse: GCCGTACTTGCCATCCTTCT
*MMP9*	Forward: GGACAAGCTCTTCGGCTTCT Reverse: TCGCTGGTACAGGTCGAGTA
*GAPDH*	Forward: TGATGACATCAAGAAGGTGGTGAAG Reverse: TCCTTGGAGGCCATGTGGGCCAT

## Data Availability

The data generated in this study can be obtained from the corresponding author upon request.

## Supplementary Materials

The following supporting information can be downloaded at: https://www.mdpi.com/article/10.3390/biomedicines13051021/s1, Figure S1: High-Magnification Fluorescence Imaging of Lipid Droplets and Lysosomal Function; Table S1: Cisplatin administration concentration. File S1. WB original data.

## Abbreviations

The following abbreviations are used in this manuscript:

TAMs	Tumor-associated macrophages
M0 macrophages	THP-1 cells after PMA stimulation
M0-control macrophages	M0 macrophages with SNX10 control expression
M0-SNX10-EO macrophages	M0 macrophages with SNX10 overexpressed
