# Supplementary material for: SNX10 Is Involved in Ovarian Cancer Cell Metastasis by Repolarizing Tumor-Associated Macrophages Through mTOR1/Lysosomes Pathway
Source: Biomedicines. 2025 Apr 23;13(5):1021. doi: 10.3390/biomedicines13051021 (PMC12109050; doi:10.3390/biomedicines13051021)

Figure 2A: The expression of SNX10 in the THP-1cell line with or without SNX10 overexpression.

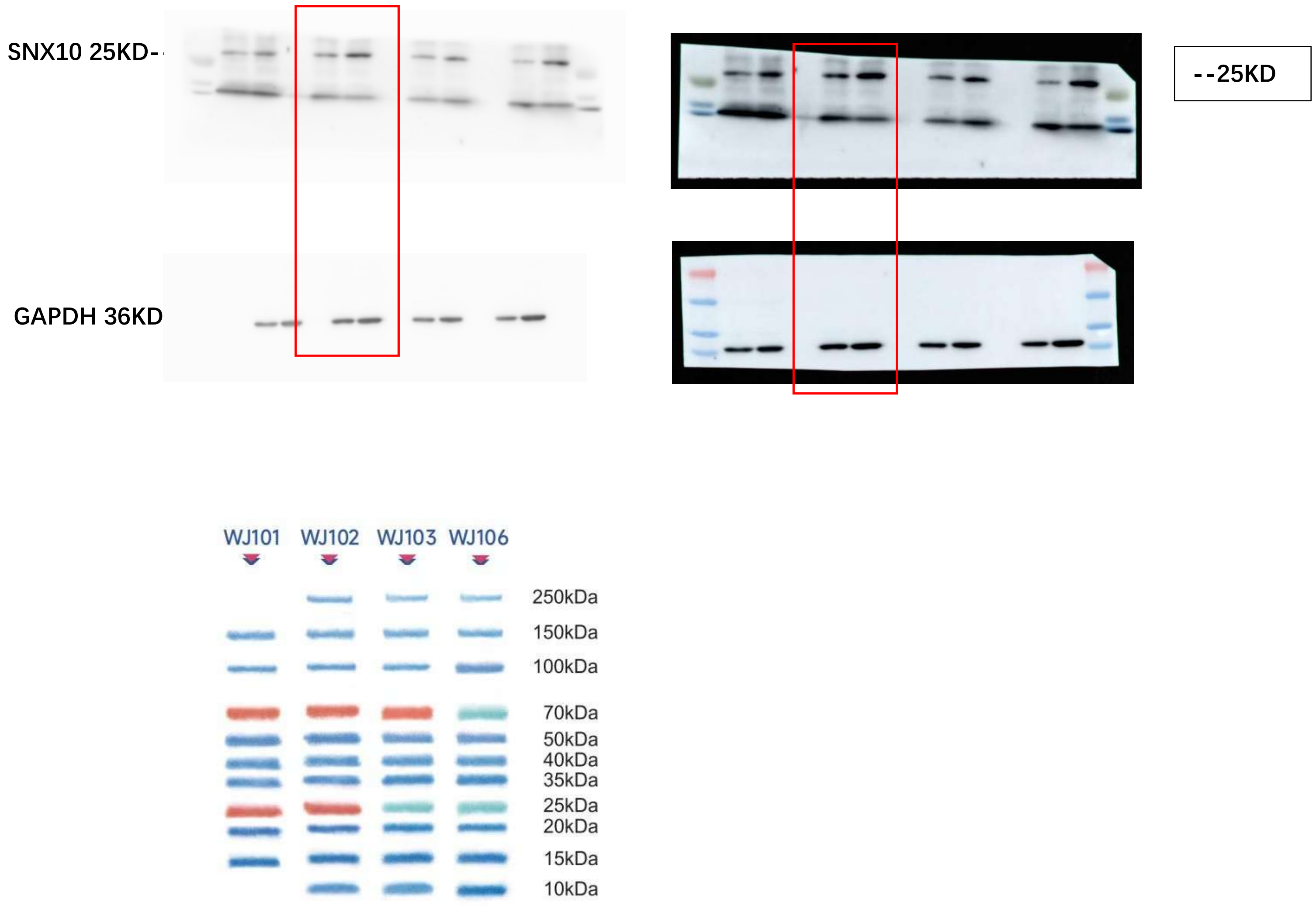

Figure 4E:The expression of p-mTORC1 in M0 macrophages after adding Everolimus or PBS for 24h

p-mTOR1 289KD--

GAPDH 36KD--

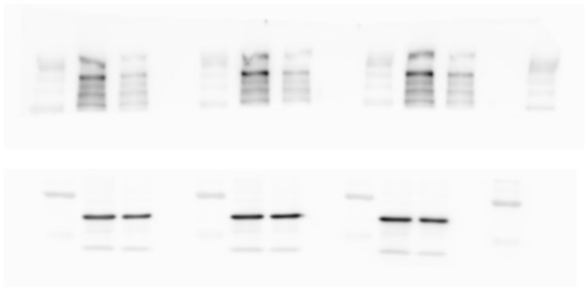

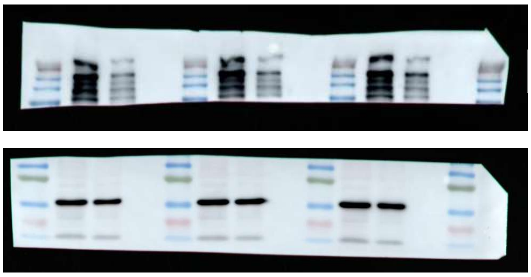

--270KD

--52KD

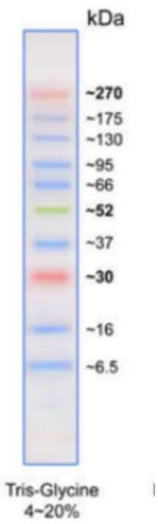

Figure 4C:The expression of p-mTORC1 and mTOR1 in THP-1 cells with or without SNX10 overexpression.

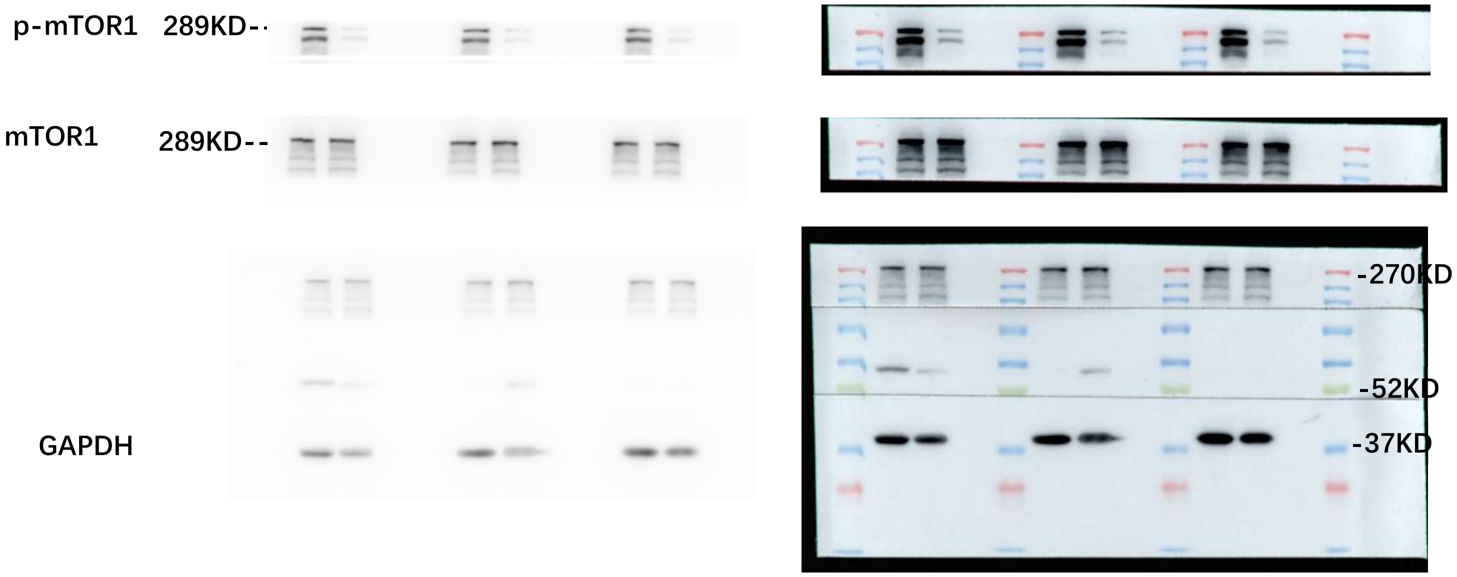

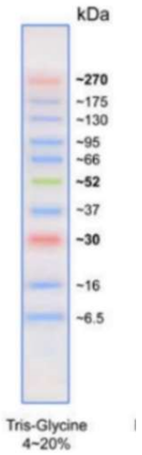

Figure 5B: The expression of LAMP2A in THP-1 cells with or without SNX10 overexpression.

LAMP2A 120

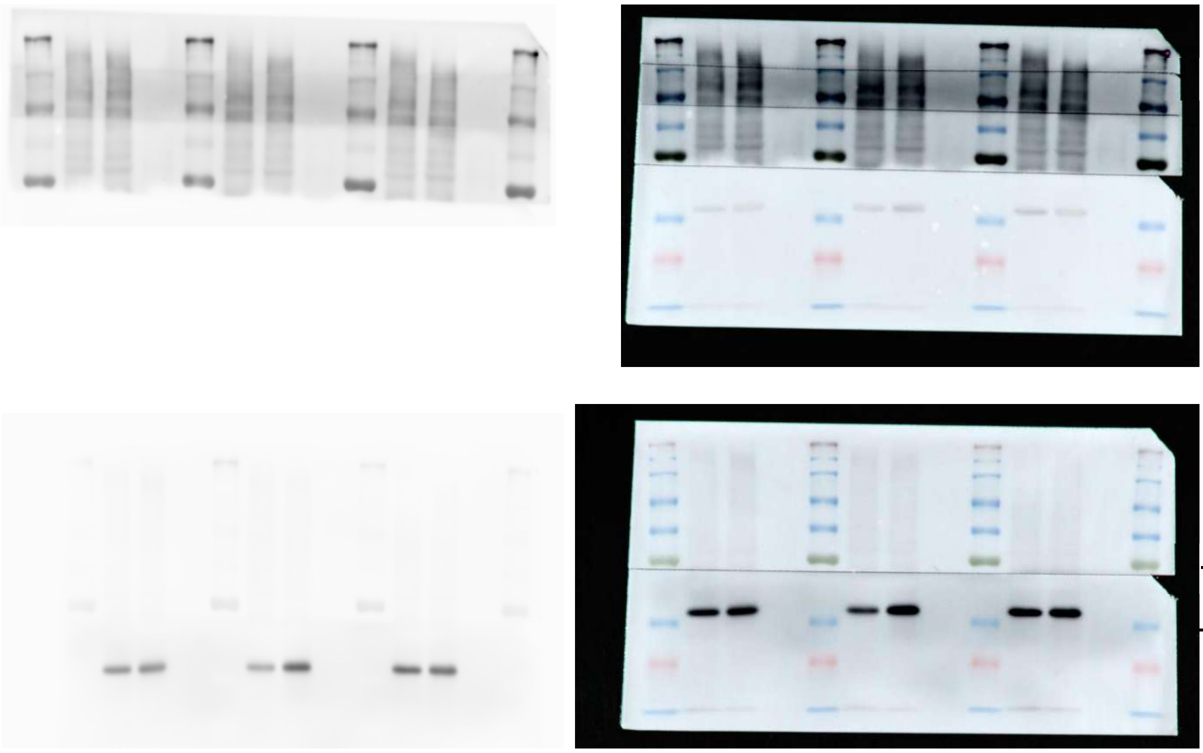

--130KD

--52KD

--37KD

GAPDH 36KD-

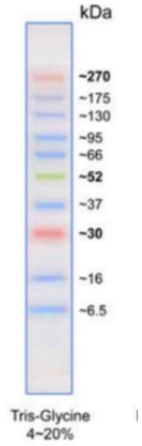

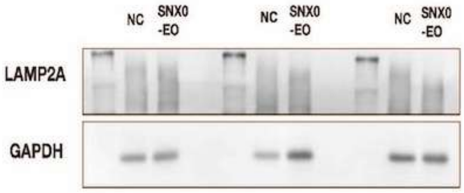

Figure 5D The expression of LAMP2A in M0 macrophages after adding Everolimus or PBS for 24h

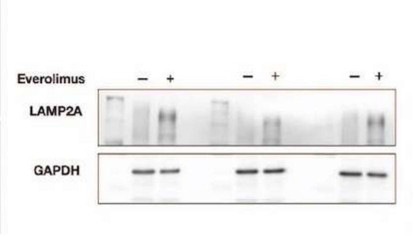

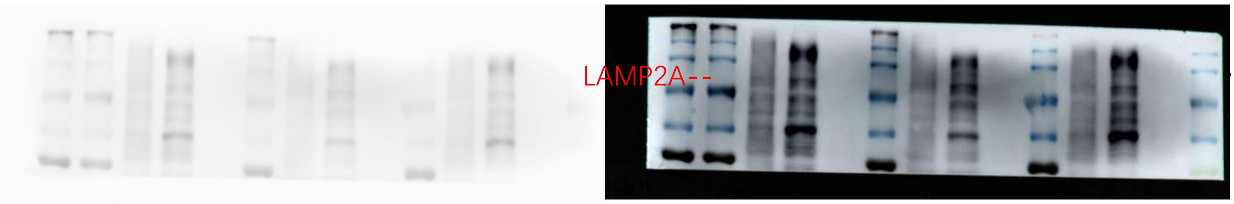

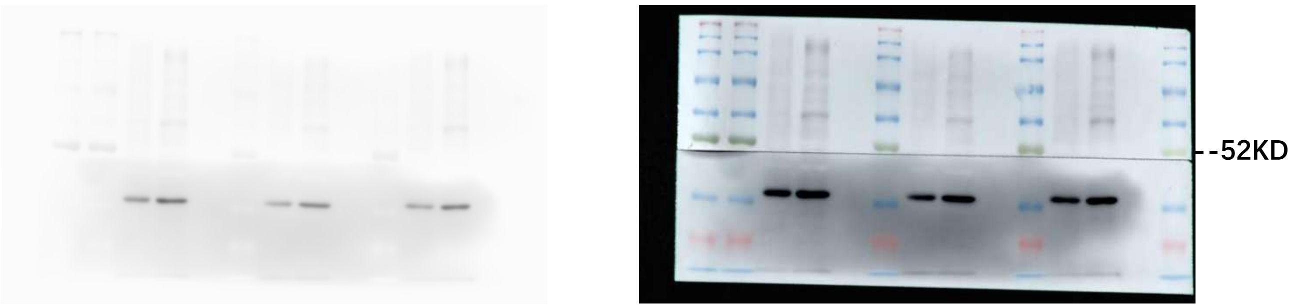

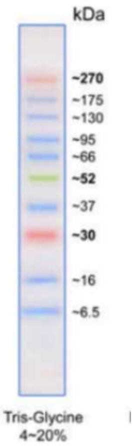

Figure 5E：The expression of p-mTORC1 and SNX10 in THP-1 cells after adding BafiomycinA1 or PBS for 12h

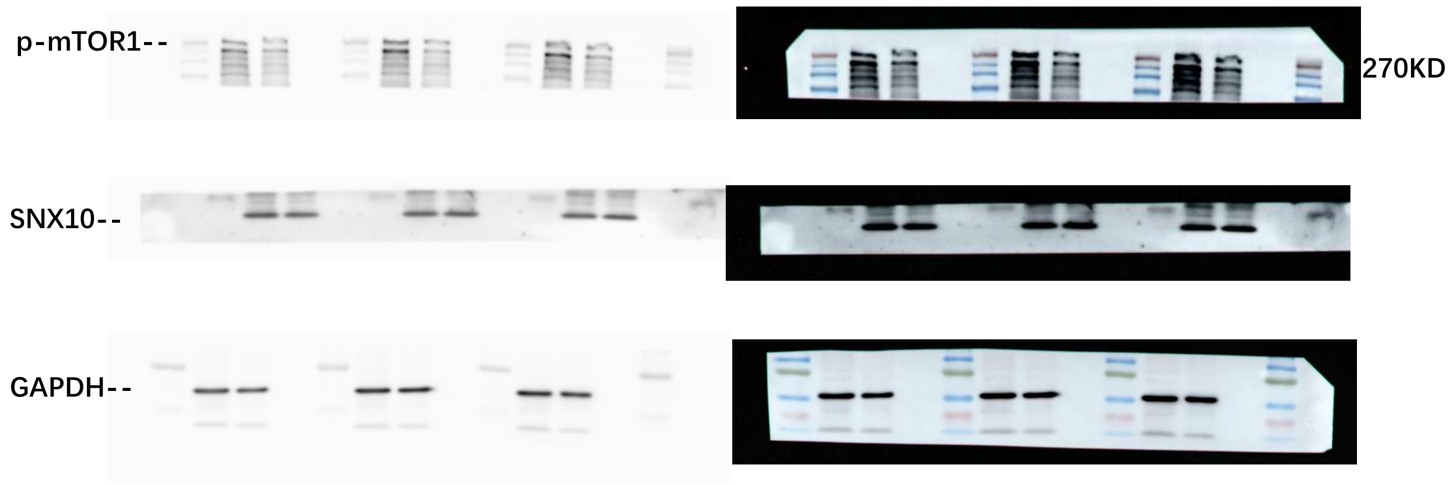

Supplement: Supplementary file 1 [file biomedicines-13-01021-s001.zip › File S1. WB original data.pdf]
